# Effect of ceramic materials and tooth preparation design on computer‐aided design and computer‐aided manufacturing endocrown adaptation and retentive strength: An in vitro study

**DOI:** 10.1002/cre2.843

**Published:** 2024-01-31

**Authors:** Ahmed Farghal, Karim Dewedar, Mohammed H. AbdElaziz, Samah Saker, Mahy Hassona, Radwan Algabri, Ahmed Yaseen Alqutaibi

**Affiliations:** ^1^ Substitutive Dental Science, College of Dentistry Taibah University Madinah Saudi Arabia; ^2^ Crown and Bridge Department, Faculty of Dental Medicine Al‐Azhar University Cairo Egypt; ^3^ Fixed Prosthodontics, Faculty of Dentistry Mansoura University Mansoura Egypt; ^4^ Prosthodontic Department, Faculty of Dentistry Ibb University Ibb Yemen; ^5^ Prosthodontic Department, Faculty of Dentistry National University, Ibb branch Ibb Yemen

**Keywords:** adaptation, endocrown, monolithic zirconia ceramic, preparation design

## Abstract

**Objectives:**

To evaluate how various tooth preparation designs impact the adaptation—both at the margins and internally—and the retentive strength of computer‐aided design and computer‐aided manufacturing (CAD/CAM) produced endocrowns.

**Materials and Methods:**

60 extracted human mandibular first molars were endodontically treated and assigned into three groups (*n* = 20) according to the tooth preparation design: Group N: butt joint design, Group F and F1 received 1‐ and 2‐mm circumferential ferrule preparation, respectively. Endocrowns were milled using either lithium disilicate glass‐ceramic (IPS emax ceramic) or monolithic zirconia. The internal and marginal adaptation of the endocrowns were evaluated using the replica technique. After cementation, the endocrowns of all test groups were dislodged axially at 0.5 mm/min using a universal testing machine. A 2‐way ANOVA and the independent samples t‐test (α = .05) were performed to statistically analyze the data.

**Results:**

The effect of changing the design of the tooth preparation (butt joint, ferrule) on the marginal and internal gap was shown to be statistically significant (*p* < .05); the lower gap values were recorded at the axial followed by cervical, marginal, and pulpal floor walls in both ceramic groups regardless of the teeth preparation design. The ANOVA test revealed similar average removal forces and stresses for the two types of tested ceramic materials.

**Conclusion:**

IPS emax ceramic adapted better than monolithic zirconia ceramic, regardless of the preparation design. Ferrule preparation design is more retentive than butt joint preparation, regardless of the type of ceramic material used.

## 1 INTRODUCTION

Restoring endodontically treated teeth with significant tooth loss poses a complex challenge involving intricate biomechanical alterations and structural considerations, for which various prosthodontic options with different designs and materials are available (Girotto et al., 2021; Ploumaki et al., 2013). Innovations in adhesive technology and an increasing focus on minimally invasive approaches in the dental field have led to alternative treatment options, including the endocrown, a monolithic composite or ceramic restoration that is fastened to the pulp chamber space and cavity margins for retention (Al‐Dabbagh, 2021; Biacchi et al., 2013; Fages & Bennasar, 2013; Sedrez‐Porto et al., 2016).

Endodontically treated teeth are conventionally restored through adhesive procedures involving the construction of a post and core, followed by the placement of full coverage crowns with an adequate ferrule; however, caution is needed when employing metal posts due to potential challenges in adaptation to the root canal geometry and an increased risk of accidental root perforation during post space creation (Girotto et al., 2021; Ploumaki et al., 2013). In contrast, endocrowns provide stability and superior resistance to fractures (Biacchi & Basting, 2012; Dietschi et al., 2008). The impressive 10‐year survival rate of 99.0% and a success rate of 89.9%, (Sedrez‐Porto et al., 2016) underscore their suitability as a treatment choice for restoring both the function and esthetics of teeth that have undergone root canal treatment (Belleflamme et al., 2017; Govare & Contrepois, 2020).

Endocrowns offer numerous advantages over post‐core and crowns, such as more straightforward preparation and less clinical time, and visits are required as many technical steps for post‐cementation, core build‐up, and possible crown lengthening are unnecessary (Al‐Dabbagh, 2021). Furthermore, it can be used when posts are contraindicated due to narrow or short canals. Nonetheless, endocrown is not indicated in cases with a narrow and short pulp chamber and if there is no sufficient remaining tooth structure (Belleflamme et al., 2017; Clausson et al., 2019; Einhorn et al., 2019; El Ghoul et al., 2020).

Although the original preparation design described by ***Bindl and Mormann*** (Bindl & Mörmann, 1999) was commonly used, some modifications to the basic preparation design were also recorded (Dogui et al., 2018; Einhorn et al., 2019). The recommended preparation design includes; 90° butt margins, cusp reduction between 2 and 3 mm, 6° internal taper of the pulpal chamber with the flat pulpal floor, and supragingival enamel margins (Einhorn et al., 2019; Hajimahmoodi et al., 2023; Magne & Knezevic, 2009; Taha et al., 2018a). It has been reported that incorporating ferrule features into the preparation of endocrowns can enhance their resistance to fracture, especially in ceramic materials (Einhorn et al., 2019).

Although lithium disilicate ceramics have gained popularity as a suitable material for various ceramic restorations due to their favorable mechanical, physical, and bonding properties, the emergence of monolithic esthetic zirconia has enabled the production of less invasive and more pleasing restorations with excellent mechanical characteristics (Alqutaibi et al., 2022; Alqutaibi, 2020; Altayyar et al., 2023; Demachkia et al., 2023; El‐Ma'aita et al., 2022).

Tooth preparation design combined with the type of materials used influences a restoration's fit and retention (Shin et al., 2017). Marginal adaptation is considered one of the main influencing factors for the longevity of any restoration. If there is a large marginal gap between the tooth and the prosthesis, the exposed luting material is dissolved in the oral environment with subsequent caries, loss of restoration retention, and ultimate failure of the prosthesis (Seo et al., 2009; Soliman et al., 2022).

Several studies assessed the influence of various materials on the fit of endocrown restorations, (El Ghoul et al., 2020; Hajimahmoodi et al., 2023) however, others evaluated the impact of preparation designs on the fit of endocrowns, (Gaintantzopoulou & El‐Damanhoury, 2016; Seo et al., 2009) but to the best authors' knowledge, no study has evaluated the impact of tooth preparation design on adaptation (marginal and internal) and retentive strength of endocrowns fabricated using different types of monolithic CAD/CAM ceramics.

This study aimed to assess the influence of tooth preparation designs on the adaptation (marginal and internal) and retentive strength of endocrown restorations fabricated from either lithium disilicate glass‐ceramic or monolithic zirconia ceramic. The null hypotheses proposed were that the tooth preparation designs or material type would not affect the adaptation and retentive strength values.

## 2 MATERIALS AND METHODS

### 2.1 Teeth selection, preparation, and grouping

Freshly extracted intact human mandibular molars (*n* = 60), caries‐free, without visible fracture lines or cracks, were chosen. The teeth were collected with no associated patient identifiers following an institutional review board. The recruited teeth have similar buccolingual (BL, 10.47 ± 1 mm) and mesiodistal (MD, 11.13 ± 1 mm) dimensions, and a maximum deviation of 10% from the average was used; the measurements were performed using a digital caliper (Insize Co.). The selected teeth were ultrasonically cleaned and kept in distilled water until use. The recruited teeth were cut horizontally above the highest point of the cementoenamel junction by 3 mm with a disc attached to the milling machine (BEGO. PARASKOP M.100‐120). The teeth were endodontically treated by the same operator (M.H) using a protaper system (Dentsply‐Maillefer) with the same sequence for standardization using sodium hypochlorite solution (2.5%) as a rinse throughout the instrumentation procedure. During the procedure, teeth were handled with gauze and stored in a saline solution between steps to prevent dehydration.

After root canal treatment, excess sealer and debris were cleaned from the walls of the access cavity of each tooth using ethylene alcohol, and the teeth were mounted in auto‐polymerizing acrylic resin (Ivocron; Ivoclar Vivadent AG) parallel to their long axis 3 mm below the cementoenamel junction to ensure the centralization and alignment of the specimen to the mold. To attain a flat restoration wall at the roof of the pulpal chamber parallel to the occlusal table of the endocrown, a two‐step, self‐etch adhesive (Clearfil SE; Kuraray America) and a dual cure core material were used according to manufacture recommendation (Einhorn et al., 2019).

**Figure 1:**
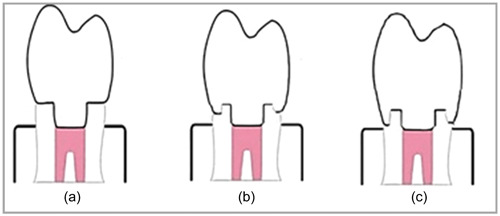
Schematic presentation of teeth preparation design used. (a) Butt joint; (b) 1 mm ferrule and (c) 2 mm ferrule.

The endodontically‐treated teeth (*n* = 60) were assigned into three main groups based on the coronal preparation type: Group N: butt joint design; no further preparation was received. Group F and F1 received 1‐ and 2‐mm circumferential ferrule preparation apical to the endocrown occlusal surface. All access cavities had approximately the same sizes with 8°–10° internal wall taper and rounded internal angles Figure 1.

**Figure 2:**
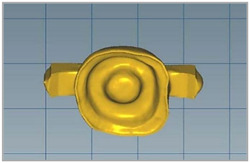
CAD of the endocrown with occlusal bar to facilitate removal after cementation.

Data from each prepared tooth were captured using an intraoral scanner TRIOS 3; 3Shape A/S. The endocrowns were designed with a 60‐μm cement space using computer‐aided design (CAD) software (3Shape CAD Design software, 3Shape). Following the manufacturer's recommendation, the endocrown restorations were milled using a CAD/CAM milling machine (Ceramill; Amann Girrbach AG). A lithium disilicate glass‐ceramic blocks (IPS e. max CAD, Ivoclar‐Vivadent) and monolithic zirconia ceramic (Zolid FX, Amann Girrbach AG) are used in this study with 2 mm occlusal thickness and were designed with a bar on the occlusal surface (measuring 15 mm by 3.5 mm by 3.5 mm) similar to a previous study (El Ghoul & Salameh, 2021). This bar was added for retention testing (i.e., for vertically removing the cemented crown from the prepared tooth, the bar served as an attachment point) Figure 2.

### 2.2 Marginal and internal adaptation assessment

Marginal and internal adaptations of the restorations were evaluated using the replica technique (El Ghoul & Salameh, 2021; Hasanzade et al., 2020; Yang et al., 2020). Each endocrown was loaded with a light‐body impression material (President light body green; Colten, Konstanz, Germany) and seated for 5 min under an axial constant force of 50 N. After the setting of light‐body material, the restoration was detached, and a heavy‐body silicone was injected into the tooth (President heavy body) to stabilize the thin silicone film. After polymerization, each replica was cut from the center into four sections in a mesiodistal and buccolingual direction using a sharp surgical blade (no. 11; Feather Safety Razor Co., Ltd.). A parallel wall slice of 2‐mm‐thickness was sectioned from each piece to facilitate the process of stereomicroscope evaluation.

The green‐colored light film presented the gap between the tooth, and the restoration was examined by a digital trinocular stereo microscope (AmScope 3.5; Irvine) at ×50 magnification. Each slice has been divided into four areas of interest for better comparison: marginal, cervical, axial, and pulpal floor (El Ghoul et al., 2020).

On each slice, eight measurements have been selected: 1 marginal gap measurement, M1; 2 cervical area measurements, C1 in the center and C2 at the cervico‐axial angle; 3 axial measurements (A1, A2, and A3) and two pulpal measurements; P1 on axio‐pulpal angle; and P2 on the center of the pulpal area.

The marginal gap was represented as M1 measurement, whereas the average of C1, C2, A1, A2, A3, P1, and P2 expressed internal adaptation of the restoration. A total of 1920 measurements were made for the six groups (eight measurements sections endocrowns × six groups).

### 2.3 Assessment of the surface area

The surface area of each preparation was estimated using an optical technique. For scanning and area measurement, a duplicate definitive die was created and digitized (3D laser scanning). Point clouds representing the prepared surface were registered, and the captured data was converted into the standard tessellation language (STL) format using a triangulation routine. Before each scan, the scanner was calibrated. Automated quality inspection and control software (Qualify 12; Geomagic GmbH) that measured the area of each triangle in the STL file was used to obtain the surface area (Johnson et al., 2018).

### 2.4 Cementation procedure

Before cementation, the prepared teeth were cleaned for 15 s using fluoride‐free pumice paste and rinsed for 15 s with water. The endocrowns were ultrasonically cleaned for 3 min in a bath containing 99% isopropanol. After cleaning, the bonding surfaces of lithium disilicate endocrowns were etched with 5% hydrofluoric acid (IPS Ceramic Etching Gel; Ivoclar Vivadent) for 20 s, rinsed for 30 s with water spray, dried with oil‐free compressed air, and silanated with a silane coupling agent (Monobond Plus; Ivoclar Vivadent) that was applied with a micro brush for two, 60‐second intervals and the excess was expelled with compressed air. The bonding surfaces of zirconia endocrowns were air abraded (Basic eco; Renfert GmbH) 110 μm aluminum oxide (Al_2_O_3_) at pressure 3 bar for 20 s at 4.0 cm distance, then cleaned for 10 min with an ultrasonic cleaner. Under finger pressure, the endocrowns were then cemented with self‐adhesive resin cement {Rely X Unicem (3 M ESPE, Seefled, Germany). A specially designed device was used to apply an axial load for 5 min with 1 kg; after removal of the excess cement, the light curing for 20 s was done for each side.

The cemented endocrowns were stored in 37℃ water for 24 h and thermocycler in a water bath with temperatures ranging between 5 and 55℃ (with a 15‐second dwell time at each temperature) for 5000 cycles before testing (Johnson et al., 2018). The specimens were subjected to thermomechanical aging, which consisted of 10,000 thermal cycles (SD Mechatronic thermocycler THE‐1100, SD Mechatronics, Westerham, Germany) in distilled water at 5 and 55℃ with a 30 s dwell time (Shokry et al., 2022).

### 2.5 Retention test

**Figure 3:**
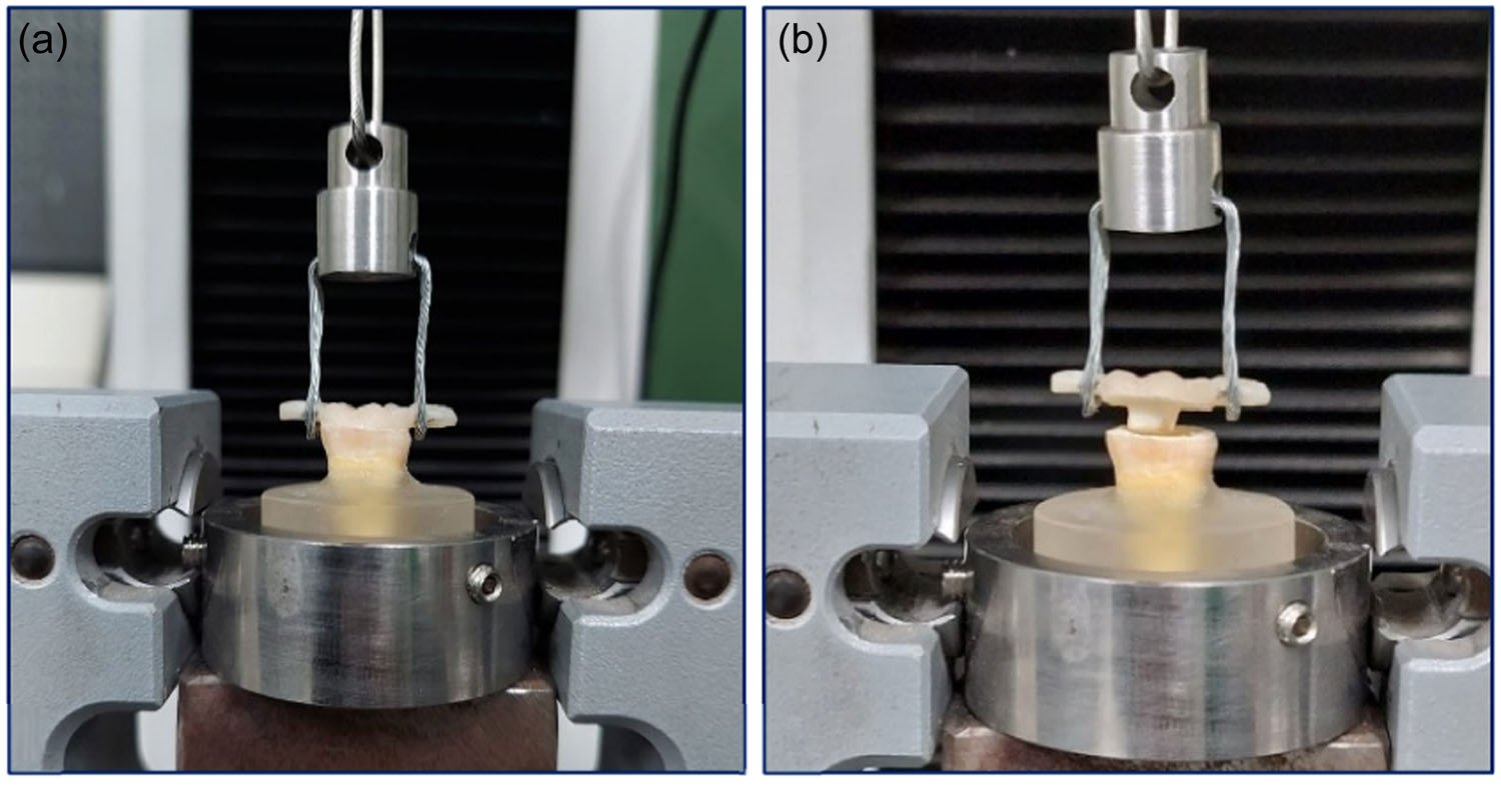
Endocrown tested for retention under universal testing machine (a), A pull‐out retention test (b).

The specimens were securely attached to the base of a universal testing machine (Instron; Instron Corp, Cantonusing tightening screws. The crown was affixed to the movable upper section using a loop wire (as depicted in Figure 3) that was fastened around the occlusal bar. The cemented crowns gradually increased vertical force along the insertion path, with the crosshead advancing at a rate of 0.5 mm per minute until the point of failure was reached. The measurement of retention strength was conducted in Mega Pascals (MPa), whereas the corresponding force necessary for dislodgement was recorded in Newtons (N). After the dislodgement of the endocrowns, the failure patterns were recorded using the criteria indicated in Table 1 after the teeth and restorations had been evaluated (Johnson et al., 1998; Palacios et al., 2006).

**Table 1:** Failure mode distribution pattern after endocrown removal.

Category	Description	Nature
**1**	Cement retained mainly (75% and more) on the prepared tooth	Adhesive
**2**	Cement retained on both endocrown and the prepared tooth (25%–75%)	Cohesive
**3**	Cement retained mainly (75% and more) on endocrown fitting surface	Adhesive
**4**	Fracture of the tooth root without endocrown separation	Cohesive

### 2.6 Statistical analysis

Shapiro‐Wilk's and Levene's tests were used to test the assumption of normal distribution of the adaptation (marginal and internal) and retention data. Given a no‐significant Levene test for equal variances, a two‐way analysis of variance (ANOVA) was used to assess the overall statistical significance of differences among study variables. To compare the marginal, cervical, axial, pulpal, and internal regions between the two tested ceramic groups, a student t‐test was used. Pairwise statistical comparison was performed using Tukey's post‐hoc test. *p* < .05 were statistically significant in all tests.

## 3 RESULTS

Regarding marginal and internal adaptation, the means (μm) and standard deviations at different locations are shown in Table 2. The lower gap values were recorded at the axial followed by cervical, marginal, and pulpal floor walls in both ceramic groups regardless of the teeth preparation design. Two‐way ANOVA revealed a significant interaction between the two independent variables (ceramic type and preparation design) on marginal gap values (*p* = .015); a nonsignificant interaction in cervical, axial, and pulpal values was reported (*p* = .212, .993, and .794 respectively) as shown in Table 3.

**Table 2:** Adaptation and group comparison of gap thickness at various locations across tested groups.

	Preparation design	Ceramic type	Mean	Std. Deviation	N	*t*‐value	*p*‐value
**Marginal**	Butt joint	IPS e. max	90.30^a^	8.47	10	−1.826	.085
		Zolid FX	98.30^a^, ^b^	10.97	10		
	1 mm ferrule	IPS e. max	101.20^b^	11.73	10	−2.747	.013
		Zolid FX	114.60^c^	10.02	10		
	2 mm ferrule	IPS e. max	95.80^b^	9.81	10	−5.937	.000
		Zolid FX	122.80^d^	10.52	10		
**Cervical**	Butt joint	IPS e. max	85.90^e^	8.52	10	−2.068	.053
		Zolid FX	94.60^a^, ^e^	10.22	10		
	1 mm ferrule	IPS e. max	88.30^a^, ^e^	9.31	10	−4.118	.001
		Zolid FX	109.00^b^, ^c^	12.88	10		
	2 mm ferrule	IPS e. max	94.50^a^, ^b^, ^e^	11.64	10	−3.200	.005
		Zolid FX	112.70^c^	13.71	10		
**Axial**	Butt joint	IPS e. max	89.46^a^, ^b^, ^e^	5.80	10	−0.400	.694
		Zolid FX	90.27^b^, ^e^	2.71	10		
	1 mm ferrule	IPS e. max	94.73^a^, ^b^, ^e^	5.48	10	−0.142	.889
		Zolid FX	95.12^a^, ^b^, ^e^	6.77	10		
	2 mm ferrule	IPS e. max	95.86^a^, ^b^, ^e^	6.40	10	−0.221	.828
		Zolid FX	96.46^a^, ^b^, ^e^	5.72	10		
**Pulpal**	Butt joint	IPS e. max	125.00^d^	14.14	10	−0.153	.880
		Zolid FX	125.90^d^	12.09	10		
	1 mm ferrule	IPS e. max	135.30^f^	10.78	10	−1.034	.315
		Zolid FX	140.10^f^	9.96	10		
	2 mm ferrule	IPS e. max	141.40^f^	11.67	10	−1.025	.319
		Zolid FX	147.30^f^	13.97	10		
**Internal**	Butt joint	IPS e. max	100.12^b^	6.30	10	−1.168	.258
		Zolid FX	103.59^b^	6.98	10		
	1 mm ferrule	IPS e. max	106.11^b^	5.40	10	−2.892	.010
		Zolid FX	114.74^c^	7.74	10		
	2 mm ferrule	IPS e. max	110.59^b^, c	4.55	10	−4.228	.001
		Zolid FX	121.45^d^	6.73	10		

*Note*: Groups with the same letter indicate no significant difference (*p* > .05).

^a^ R Squared = 0.570 (Adjusted R Squared = 0.531).

^b^ R Squared = 0.470 (Adjusted R Squared = 0.420).

^c^ R Squared = 0.208 (Adjusted R Squared = 0.135).

^d^ R Squared = 0.331 (Adjusted R Squared = 0.269).

^e^ R Squared = 0.583 (Adjusted R Squared = 0.544).

^f^ Computed using *α* = .05.

**Table 3:** Two‐way ANOVA test for impact of preparation design and material brand on adaptation values at different locations.

Source	Dependent variable	Type III sum of squares	df	Mean Square	F	Sig.
Corrected Model	Marginal	7608.933^a^	5	1521.787	14.338	.000
	Cervical	5999.000^b^	5	1199.800	9.559	.000
	Axial	450.880^c^	5	90.176	2.841	.024
	Pulpal	3969.933^d^	5	793.987	5.335	.000
	Inter	3058.639^e^	5	611.728	15.075	.000
Intercept	Marginal	646881.667	1	646881.667	6094.778	.000
	Cervical	570375.000	1	570375.000	4544.150	.000
	Axial	526219.350	1	526219.350	16578.769	.000
	Pulpal	1107041.667	1	1107041.667	7438.685	.000
	Inter	718552.399	1	718552.399	17707.960	.000
Preparation design	Marginal	2746.133	2	1373.067	12.937	.000
	Cervical	1821.900	2	910.950	7.257	.002
	Axial	445.039	2	222.520	7.011	.002
	Pulpal	3676.633	2	1838.317	12.352	.000
	Inter	2035.579	2	1017.790	25.082	.000
Ceramic type	Marginal	3904.267	1	3904.267	36.785	.000
	Cervical	3776.267	1	3776.267	30.085	.000
	Axial	5.400	1	5.400	.170	.682
	Pulpal	224.267	1	224.267	1.507	.225
	Inter	879.215	1	879.215	21.667	.000
Preparation design * ceramic type	Marginal	958.533	2	479.267	4.516	.015
	Cervical	400.833	2	200.417	1.597	.212
	Axial	0.441	2	.221	.007	.993
	Pulpal	69.033	2	34.517	.232	.794
	Inter	143.845	2	71.922	1.772	.180
Error	Marginal	5731.400	54	106.137		
	Cervical	6778.000	54	125.519		
	Axial	1713.990	54	31.741		
	Pulpal	8036.400	54	148.822		
	Inter	2191.208	54	40.578		

^a^ R Squared = 0.570 (Adjusted R Squared = 0.531).

^b^ R Squared = 0.470 (Adjusted R Squared = 0.420).

^c^ R Squared = 0.208 (Adjusted R Squared = 0.135).

^d^ R Squared = 0.331 (Adjusted R Squared = 0.269).

^e^ R Squared = 0.583 (Adjusted R Squared = 0.544).

A disparity in the estimated average of the gap values was noted across different sites in the butt joint preparation design. Specifically, the marginal, axial, and cervical regions exhibited similar proximity, while the pulpal regions displayed a much greater value. After implementing the ferrule preparation design, specifically using 1 mm and 2 mm ferrules, the findings indicate a notable rise in the estimated means of gap values at different locations when compared to the butt joint preparation design. The difference between the marginal and cervical regions remains consistent, with the pulp floor exhibiting the highest mean gap value, as shown in Table 2.

Irrespective of the variables being tested, such as preparation design and ceramic type, it was observed that the pulpal floor wall exhibited the highest average gap in all groups. On the other hand, the marginal, axial, and cervical walls displayed a similar level of discrepancy, which was found to be statistically significant when compared to the pulpal floor wall, as indicated in Table 2. Similarly, irrespective of the specific ceramic material employed, the average marginal gap exhibited no significant difference compared to the internal gap between groups N and F1, as indicated by the p‐values of 0.085 and 0.396, respectively. Nevertheless, within group F1, the average marginal gap exhibited a reduced magnitude compared to the internal gap observed in the IPS e. max group (*p* < .001) as presented in Table 4.

**Table 4:** Mean values (µm), standard deviations, and group comparison of marginal and internal gap thickness across tested groups.

Preparation design	Ceramic type	Adaptation	Mean	Std. Error	95% confidence interval	
					Lower bound	Lower bound
**Butt**	IPS e. max	marginal	90.30^a^	2.708	84.93	95.67
		internal	100.12^a^, ^b^	2.708	94.75	105.49
	Zolid FX	marginal	98.30^a^	2.708	92.93	103.67
		internal	103.59^b^	2.708	98.22	108.96
**1 mm ferrule**	IPS e. max	marginal	101.20^a^, b	2.708	95.83	106.57
		internal	106.11^b^	2.708	100.74	111.48
	Zolid FX	marginal	114.60^b^	2.708	109.23	119.97
		internal	114.74^b^	2.708	109.37	120.11
**2 mm ferrule**	IPS e. max	marginal	95.80^a^	2.708	90.43	101.17
		internal	110.59^b^	2.708	105.22	115.96
	Zolid FX	marginal	122.80^c^	2.708	117.43	128.17
		internal	121.45^c^	2.708	116.08	126.82

*Note*: Groups with the same letter indicate no significant difference (*p*  >  .05).

^a^ R Squared = 0.570 (Adjusted R Squared = 0.531).

^b^ R Squared = 0.470 (Adjusted R Squared = 0.420).

^c^ R Squared = 0.208 (Adjusted R Squared = 0.135).

^d^R Squared = 0.331 (Adjusted R Squared = 0.269).

^e^R Squared = 0.583 (Adjusted R Squared = 0.544).

^f^Computed using *α* = .05.

The mean and standard deviation of removal force (N), surface area (mm), and retentive strength (MPa) are presented in Table 5. Two‐way ANOVA showed a significant impact of the tooth preparation design on endocrown removal force. However, ceramic type revealed a nonsignificant impact on the removal force of endocrowns restorations Table 6. Multiple comparisons of the tooth preparation group with the Tukey test revealed that the 2‐mm ferrule groups offered statistically significantly higher retention than the butt joint group (*p* < .001).

**Table 5:** Mean values and standard deviations of removal force (N), and retentive strength (MPa) across tested groups.

	Design	Ceramic	Mean	Std. Deviation	*N*	*t*‐value	*p*‐value
Removal Force	Butt joint	IPS e. max	387.00^a^	51.43	10	−0.983	.338
		Zolid FX	362.00^a^	61.78	10		
	1 mm ferrule	IPS e. max	583.00^b^	23.84	10	−1.059	.303
		Zolid FX	561.60^b^	59.26	10		
	2 mm ferrule	IPS e. max	697.00^c^	36.53	10	−1.061	.303
		Zolid FX	677.50^c^	45.17	10		
		Zolid FX	152.50^B^	9.79	10		
Retention	Butt joint	IPS e. max	3.61^AB^	0.59	10	−0.953	.353
		Zolid FX	3.35^AB^	0.61	10		
	1 mm ferrule	IPS e. max	4.20^AB,CD^	0.20	10	−0.961	.349
		Zolid FX	4.02^AB^	0.53	10		
	2 mm ferrule	IPS e. max	4.60^CD^	0.52	10	−0.413	.685
		Zolid FX	4.51^CD^	0.48	10		

*Note*: Groups with the same letter indicate no significant difference (*p*  >  .05).

**Table 6:** Two‐way ANOVA test for impact of preparation design and material brand on adaptation values at different locations.

Source	Dependent Variable	Type III Sum of Squares	df	Mean Square	*F*	Sig.
**Corrected model**	Removal force	1008322.083^a^	5	201664.417	86.939	.000
	Surface area	20277.083^b^	5	4055.417	74.389	.000
	Retention	12.213^c^	5	2.443	9.579	.000
**Intercept**	Removal force	17800796.017	1	17800796.017	7674.049	.000
	Surface area	1064268.017	1	1064268.017	19521.884	.000
	Retention	982.450	1	982.450	3852.652	.000
**Preparation design**	Removal force	1001006.033	2	500503.017	215.770	.000
	Surface area	20264.133	2	10132.067	185.853	.000
	Retention	11.697	2	5.848	22.934	.000
**Ceramic type**	Removal force	7238.017	1	7238.017	3.120	.083
	Surface area	0.817	1	0.817	0.015	.903
	Retention	0.449	1	0.449	1.760	.190
Preparation design * ceramic type	Removal force	78.033	2	39.017	0.017	.983
	Surface area	12.133	2	6.067	0.111	.895
	Retention	0.067	2	0.034	0.132	.877
**Error**	Removal force	125258.900	54	2319.609		
	Surface area	2943.900	54	54.517		
	Retention	13.770	54	0.255		

^a^ R Squared = 0.890 (Adjusted R Squared = 0.879).

^b^ R Squared = 0.873 (Adjusted R Squared = 0.861).

^c^ R Squared = 0.470 (Adjusted R Squared = 0.421).

^d^Computed using *α* = .05.

**Figure 4:**
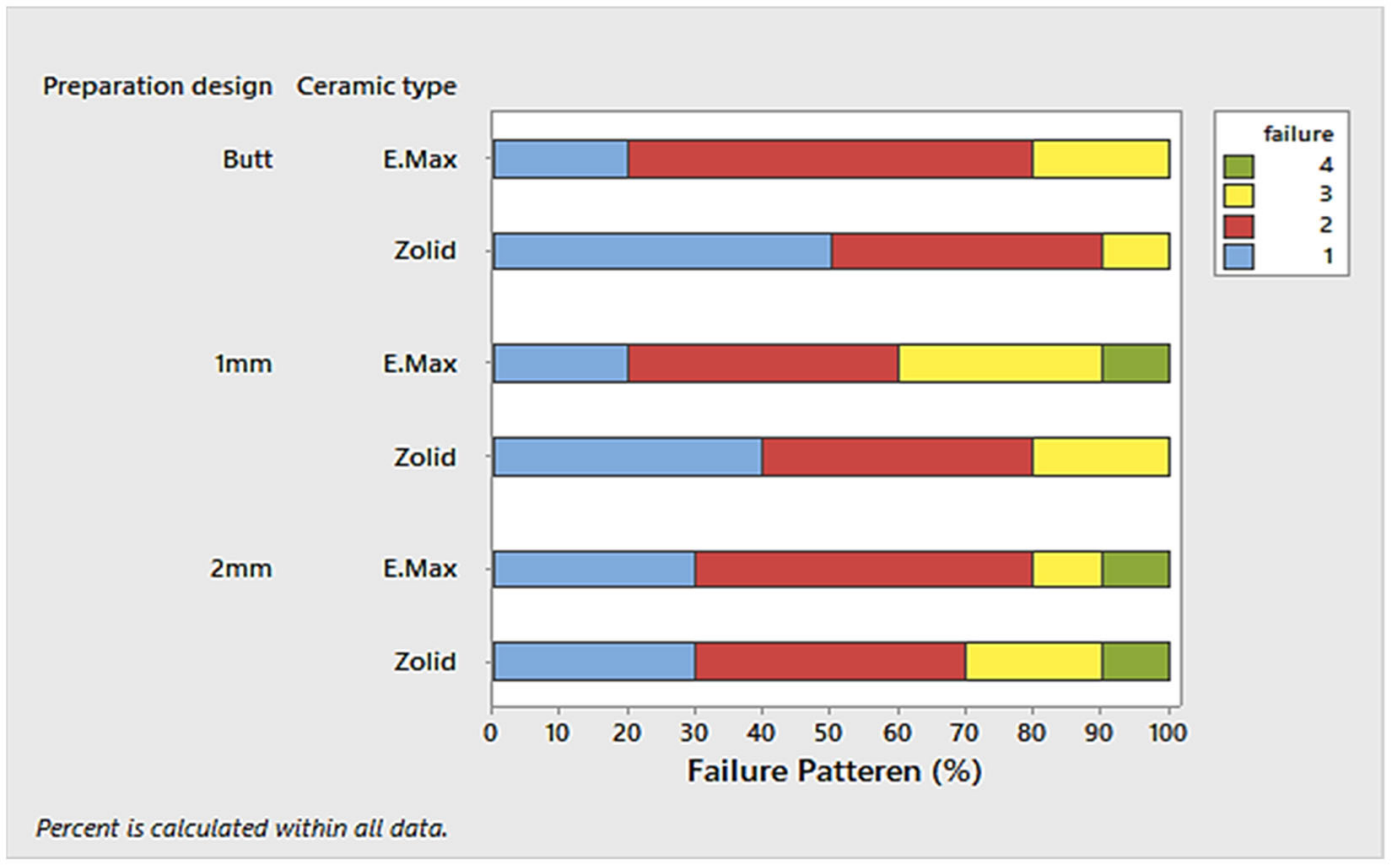
Tablet‐form figure shows the different failure modes observed following endocrown dislodgment.

The percentage of the failure pattern of the tested variables is presented in Figure 4. The main failure mode for two tested ceramic materials was cement retained on the endocrowns restorations. A mixed type of failure with a cement retained on the tooth's surface and in the endocrown was detected for all the tested groups (Figure 4).

## 4 DISCUSSION

The present study evaluated the impact of various tooth preparation designs on adaptation (marginal and internal) and the retentive strength of endocrown restorations. The results of this study showed that both the preparation design and ceramic type influenced endocrowns' adaptation values; however, the retentive strength of the endocrowns restorations had been influenced by tooth preparation design only, and the impact of ceramic type was insignificant, so the study null hypothesis was partially rejected.

A nondestructive replica technique (RT) has been chosen to evaluate the adaptation of endocrowns. The RT has been selected to evaluate marginal and internal discrepancies by previous studies (El Ghoul & Salameh, 2021; Hasanzade et al., 2020; Yang et al., 2020). Although this evaluation method is noninvasive, straightforward, reliable, less costly, and can be replicated easily without losing accuracy. The disadvantages of this method lie in the accuracy of the used impression material, measuring protocol, and insufficient number of measurements (Falk et al., 2015; Groten et al., 2000). For a reliable assessment of the entire adaptation in the current study, thirty‐two measurements per each endocrown were used. In addition, impression material was selected to capture accurate gap values as it exhibited outstanding dimensional stability.

The mean marginal and internal gaps reported in this study were 90.3–122.8 µm for all the test groups, which has been considered clinically acceptable based on the records of the previous reports. Based on those previous records, an internal and marginal gap of 75–160 mm is acceptable (El Ghoul et al., 2020; Hasanzade et al., 2021; Taha et al., 2018b).

The butt joint configuration, positioned parallel to the occlusal plane, produces a stable surface capable of withstanding compressive stresses. The forces in question are distributed compressively around the cervical butt joint or along the axial walls in a shear force manner. From a biomechanical perspective, the bonded joint of the restoration facilitates the ability to adjust to strains, enhancing the overall performance (Taha et al., 2018a).

The current study found significant differences between the two types of ceramic materials regarding their adaptation. Furthermore, the results indicated a negative relationship between material hardness and the level of adaptation observed). E. max ceramic showed better adaptation regardless of the preparation design. This result corresponds to previous reports, (El Ghoul et al., 2020; Taha et al., 2018b), which relate material machinability to their structural compositions and mechanical properties. Furthermore, a study conducted by El Ghoul (El Ghoul et al., 2020) reported superior adaptation of glass‐ceramic materials based on their mechanical characteristics.

On the other hand, implementing a 1 mm ferrule preparation design may have reduced shear stresses exerted on the axial walls and promoted a more substantial load distribution along the margins (Taha et al., 2018a). Furthermore, the decrease in axial wall dimensions might result in a reduction in resin cement thickness relative to the bulk of ceramic material compared to the butt joint preparation design. This reduction in thickness leads to a drop in both heat and polymerization shrinkage of the resin cement (Magne et al., 1999).

In this study, to assess internal adaptation in more detail, the measurement area was allocated at three different positions for better comparisons: cervical (C), axial (A), and pulpal (P) (El Ghoul & Salameh, 2021; El Ghoul et al., 2020). In all the test groups, especially in group F1, the most significant gap has consistently been observed at the pulp floor. These results were comparable to previous reports (Hasanzade et al., 2021; Taha et al., 2018b) and may refer to a little preparation convergence angle and the restricted optical depth of the scanner, resulting in a blurred pulpal region image. Additionally, the shrinkage of milled lithium disilicate blocks by about 0.3%. as a result of the crystallization procedure (El Ghoul & Salameh, 2021).

Clinically, the two factors closely concerned with plaque retention, periodontal diseases, cement dissolution, and recurrent caries are marginal and cervical adaptation of the indirect restoration (Hasanzade et al., 2020). The cervical walls displayed the smallest mean gap in the e. max group, while the axial wall displayed the smallest mean gap in the zirconia group regardless of the tooth preparation design.

Our finding supported the conclusion of Goujat et al (Goujat et al., 2019)., who stated that adapting the restoration to a non‐retentive preparation design was better. However, the findings of Yang et al (Yang et al., 2020). assessed the adaptation of CAD/CAM fabricated composite onlay fabricated using two preparation designs. They concluded that the traditional preparation design (more retentive) recorded superior adaptation.

Taha et al (Taha et al., 2018a). conducted a study that demonstrated the positive effect of a 1‐mm shoulder finish line in enhancing the fracture resistance of endocrowns. However, in another study, a 2‐mm ferrule design had a good outcome compared to other designs (Mohammed et al., 2023). There is limited knowledge regarding the influence of preparation design, particularly on both adaptation (marginal and internal) and retentive strength.

In this study, regardless of the specific ceramic material type employed, the average removal forces increased in the group with a ferrule preparation design. Although the endocrowns retention is based on macro and micromechanical retention by being affixed to the internal part of the pulp chamber and the margins of the cavity, the impact of tooth preparation design was significant. While there is a more residual tooth structure and less bonding area in a butt joint preparation design, a 90‐degree shoulder preparation displayed a greater bonding area and less remaining tooth tissue (Einhorn et al., 2019).

The results regarding the failure pattern provided insights into the adhesion of the cement to both the tooth structure and the endocrown material. Furthermore, additional information was reported regarding the cohesive strength of the cement (Johnson et al., 2018). In the current study, the cement was located mainly on the fitting surface of the endocrown for all test groups, showing that the cement adherence to the fitting surface of the endocrown is greater than that of the bonded dentin.

Based on the marginal preparation design of the restored teeth, the enamel layer distribution determines the bonding surface composition and the residual amount of dental tissue. While there is a more residual tooth structure and less bonding area in a butt joint preparation design, a 90‐degree shoulder preparation displayed a greater bonding area and less remaining tooth tissue (Taha et al., 2018a).

The findings of our investigation indicate that the preparation design had no significant impact on the internal fit and marginal gap. However, considerable differences have been observed in cycle fatigue tests following cyclic loading, as reported by Comba et al (Comba et al., 2022). Additional research is required to comprehensively assess the impact of material type and preparation design on the internal fit and marginal gap, specifically through the incorporation of cyclic fatigue testing.

The limitations of the present study include the study's in vitro design, which implies that the observed outcomes might not perfectly translate to real‐world clinical scenarios. The strength and rigidity of the used materials and their resistance to cyclic loading, aging, durability, abrasion, wear, and so forth are the determinants of the restoration durability. So, further clinical long‐term trials are recommended to assess the impact of tooth preparation design, using different ceramic materials and luting cements on the clinical outcome of endocrowns restorations. Furthermore, the absence of comparisons with other types of restorations, both with and without posts, restricts the ability to draw broader conclusions about the relative efficacy of the evaluated indirect restorations. Future research endeavors could incorporate such comparative analyses to provide a more nuanced and comprehensive assessment of different restoration approaches.

## 5 CONCLUSIONS

Within the limitations of this in vitro study, it was concluded that:

1. Using butt joint preparation design results in better adaptation (lesser marginal and internal gaps) of CAD/CAM endocrowns compared to 1 and 2 mm‐ferrule preparation, regardless of the ceramic material used.
2. IPS emax ceramic adapted better than monolithic zirconia, regardless of the tooth preparation design.
3. Ferrule preparation design is more retentive than butt joint preparation, regardless of the type of ceramic material used.
4. Additional studies are needed to assess the retention force of Zirconia endocrowns with varied surface treatments and adhesive systems under cyclic loading and thermocycling while also recommending extended clinical trials to evaluate the impact of tooth preparation design, diverse ceramic materials, and luting cement on the long‐term clinical outcomes of endocrown restorations.

## AUTHOR CONTRIBUTIONS

**Ahmed Farghal, Samah Saker, Ahmed Yaseen Alqutaibi, and Mohammed H. AbdElaziz**: Conceptualization; study design; methodology; data interpretation; data analysis. **Samah Saker, Mohammed H. AbdElaziz, Karim Dewedar, Mahy Hassona and Radwan Algabri**: Resources; writing the manuscript. The manuscript was reviewed by all of the writers. The final version of the manuscript was read and approved by all authors.

## CONFLICT OF INTEREST STATEMENT

The authors declare no conflicts of interest.

## ETHICS STATEMENT

The research protocol received approval from the Faculty of Dentistry ethics committee at Taibah University, with the assigned reference number 210323. All methodologies employed in this investigation strictly adhered to the pertinent standards and laws.

## Data Availability

The datasets utilized in this investigation can be obtained from the corresponding author upon a reasonable request.
